# Microbiome–metabolome reveals the contribution of gut–kidney axis on kidney disease

**DOI:** 10.1186/s12967-018-1756-4

**Published:** 2019-01-03

**Authors:** Yuan-Yuan Chen, Dan-Qian Chen, Lin Chen, Jing-Ru Liu, Nosratola D. Vaziri, Yan Guo, Ying-Yong Zhao

**Affiliations:** 1 School of Pharmacy, Faculty of Life Science & Medicine, Northwest University, No. 229 Taibai North Road, Xi’an, 710069 Shaanxi China; 2 Division of Nephrology and Hypertension, School of Medicine, University of California Irvine, Irvine, CA 92897 USA; 3 Department of Internal Medicine, University of New Mexico, Albuquerque, 87131 USA

**Keywords:** Microbiome, Gut microbiota, Metabolome, Kidney diseases, Probiotics

## Abstract

Dysbiosis represents changes in composition and structure of the gut microbiome community (microbiome), which may dictate the physiological phenotype (health or disease). Recent technological advances and efforts in metagenomic and metabolomic analyses have led to a dramatical growth in our understanding of microbiome, but still, the mechanisms underlying gut microbiome–host interactions in healthy or diseased state remain elusive and their elucidation is in infancy. Disruption of the normal gut microbiota may lead to intestinal dysbiosis, intestinal barrier dysfunction, and bacterial translocation. Excessive uremic toxins are produced as a result of gut microbiota alteration, including indoxyl sulphate, *p*-cresyl sulphate, and trimethylamine-N-oxide, all implicated in the variant processes of kidney diseases development. This review focuses on the pathogenic association between gut microbiota and kidney diseases (the gut–kidney axis), covering CKD, IgA nephropathy, nephrolithiasis, hypertension, acute kidney injury, hemodialysis and peritoneal dialysis in clinic. Targeted interventions including probiotic, prebiotic and symbiotic measures are discussed for their potential of re-establishing symbiosis, and more effective strategies for the treatment of kidney diseases patients are suggested. The novel insights into the dysbiosis of the gut microbiota in kidney diseases are helpful to develop novel therapeutic strategies for preventing or attenuating kidney diseases and complications.
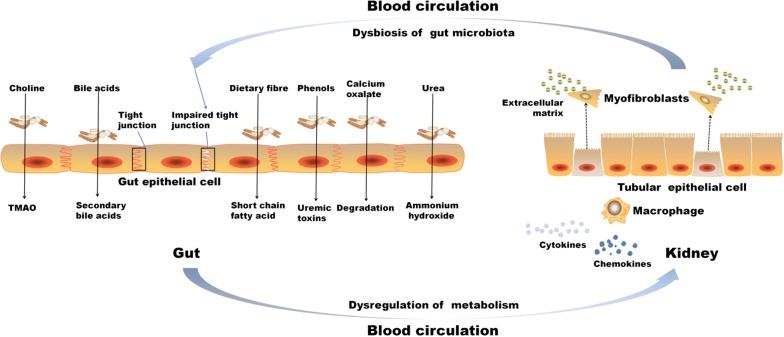

## Background

**Fig. 1:**
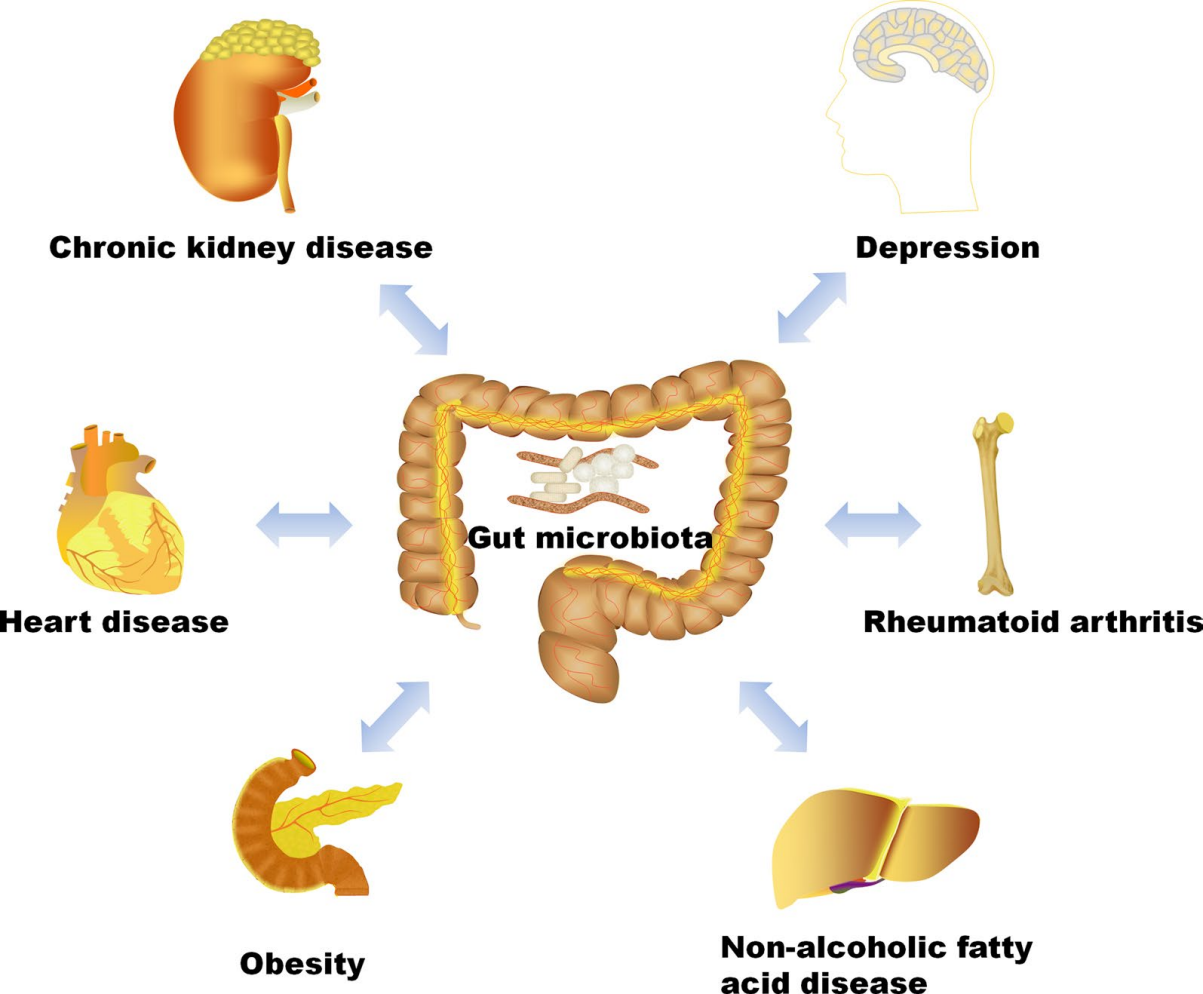
The contribution of the dysbiosis of gut microbiome on various diseases. Gut microbiome alterations and the leaky gut epithelial barrier are associated with chronic kidney disease, heart disease, obesity, non-alcoholic fatty acid disease, rheumatoid arthritis and depression

The microbiota in healthy human intestines is a complex community of more than 100 trillion microbial cells among which are more than 1000 different species [1]. In the healthy state, these microbes live in a commensal relationship with their host, modulating the immune system, protecting against pathogens, and regulating endogenous metabolism of carbohydrates and lipids, thus contributing to the nutritional balance [2]. The alterations in the microbiome are increasingly linked to the development of various diseases such as obesity, cancer, diabetes, inflammatory bowel disease, cardiovascular disease, and kidney disease [3]. Figure 1 presents the dysbiosis of gut microbiome on the influence of various diseases. Dysbiosis in gut microbiota has been implicated in the progression of various kidney diseases [4–10]. In fact, dysbiosis is often observed in uremic states characteristic of retention of uremic toxins, most of which derive from the imbalanced fermentation of nitrogen metabolites. These uremic toxins contribute to the progression and complications of CKD [11–15].

This review focuses on the pathogenic association between gut microbiota and kidney diseases (the gut–kidney axis), touching on CKD, hemodialysis, peritoneal dialysis, immunoglobulin A nephropathy (IgAN), nephrolithiasis, hypertension and acute kidney injury (AKI) patients. As we reflect on the relevant studies and summarize the accumulating findings, we come to a notation that prebiotics and probiotics as well as their combination are important adjuvant therapies for CKD treatment. Dysbiotic gut microbiota provides a potential therapeutic target for preventing or harnessing complications.

## Application of gut microbiome–metabolome approaches to the study of gut microbiota

**Fig. 2:**
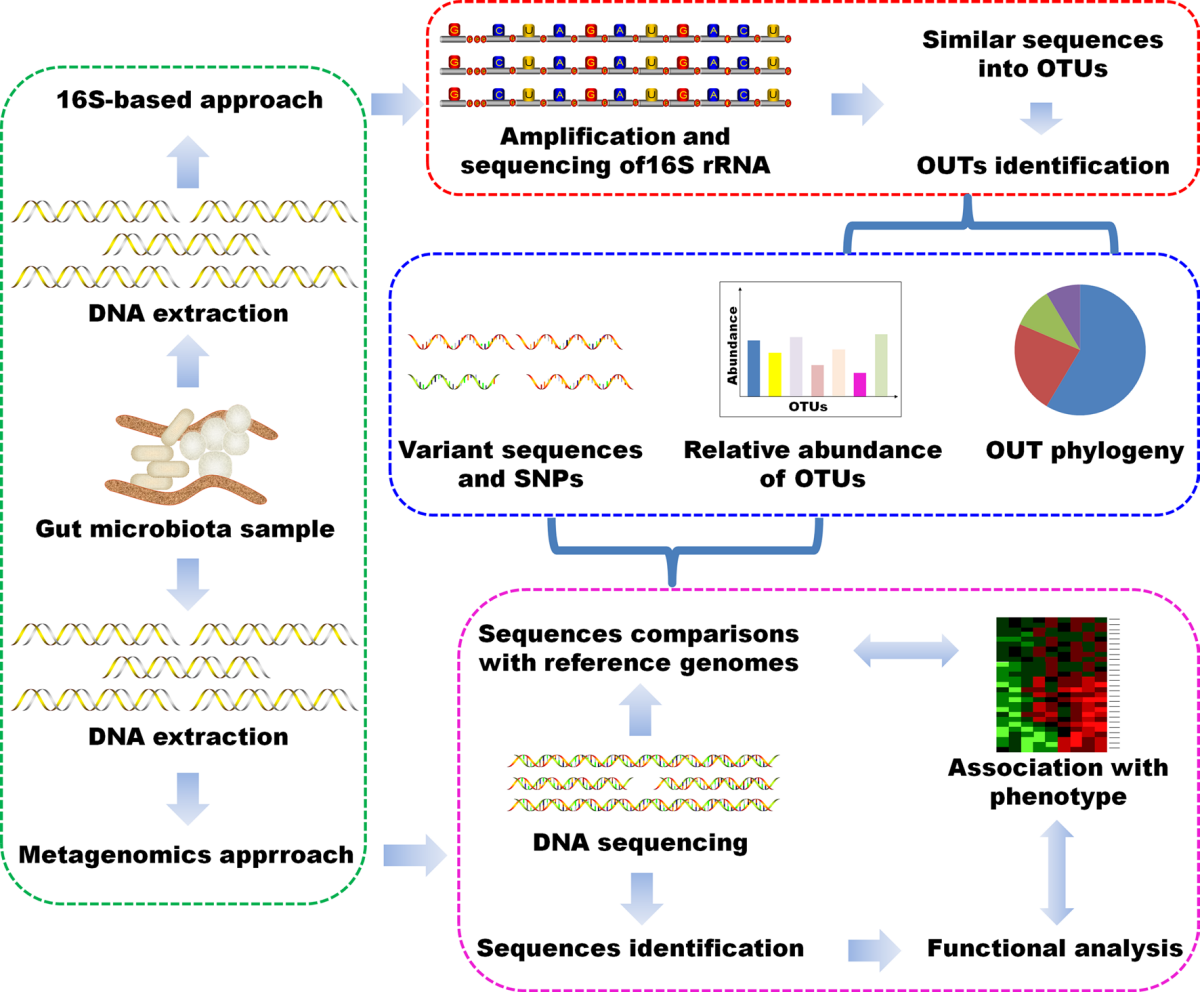
Work flows for 16S-based and metagenomics approaches. Microbial community samples contain various species of bacteria and other microorganisms, here indicated by different colors and shapes. After total DNA extraction, the community composition was detected by amplifying and sequencd the 16S rRNA gene. Highly similar sequences are grouped into OTUs, which were labeled by comparison with databases of recognized organisms. OTUs provided the presence/absence, abundance, or phylogenetic diversity. The total metagenomic DNA may be sequenced and compared with function-oriented databases to analyze biomolecular and metabolic functions present in the community. Additionally, sequenced community DNA can be compared with reference genomes. These can identify microbial sequence variants and polymorphisms and provides an alternative method of determining the presence and abundance of specific organisms

Establishment of advanced next-generation sequencing technologies, including metagenomics and 16S ribosomal RNA (rRNA) sequence analysis has facilitated the analysis of a much larger number of gut microorganisms. Both approaches have their own unique advantages. Metagenomic sequencing is aimed at determining “what they can do” by random sequencing all extracted DNA in the sample [16], whereas the 16S rRNA analysis was more useful in finding “who’s there?” by sequencing the conserved 16S rRNA gene that present in all bacteria [17]. Functional analysis by shotgun metagenomics is highly dependent on our underlying knowledge of how gene sequences code for enzymatic or other functions, and metabolic databases such as KEGG and MetaCyc are great resources in this respect. Figure 2 summarizes some methodologies used to the study of microbiome. Despite some advances in microbiome-sequencing workflows, gut microbiome research is faced with many challenges. The limited understanding of microbial function in disease causality severely impedes generating hypotheses regarding complex mechanistic links between gut microbiome and diseases. The metabolomics could provide some important information in gut microbiome.

Metabolomics was defined as “the quantitative measurement of the dynamic multiparametric metabolic response of living organisms to pathophysiological stimulation or genetic modifications” [18–21]. As an important tool for understanding function of gut microbiota, metabolomics has emerged as a systematic approach to low-molecular-weight endogenous metabolites and can examine their changes following disease, toxic exposure, or genetic variation [22–24]. Proton nuclear magnetic resonance spectroscopy and mass spectrometry-based approach are major analytical tools for metabolomic research [24, 25]. As a powerful analytical platform, recently, metabolomics has been widely applied to facilitate various diseases’ diagnosis and prognosis, biomarker discovery, pharmaceutical development, and drug efficacy/toxicity evaluation [26–31]. Metabolomics has been widely used in studies of various kidney diseases [18–20]. Nevertheless, the application of metabolomics on gut microbiome-influenced samples from kidney diseases is rare. Such study is essential for understanding the links between gut microbiota and kidney diseases.

Overall, the infancy in both gut microbiome and metabolome data calls for the need to further our understanding mechanisms and phenotypes in links between gut microbiota and kidney diseases through multi-omics research.

## The crosstalk underlying gut–kidney axis

### Gut microbiome as a potential source of uremic toxins

Uremic toxins are traditionally categorized based on the physicochemical characteristics affecting their clearance during dialysis. These contained low water-soluble molecules (molecular weight < 500 Da), larger middle molecules (molecular weight > 500 Da), and protein-bound molecules. Uremic toxins also can be classified based on their site of origin: endogenous (mammalian metabolism), exogenous (diet) or microbial. Currently, known gut-derived uremic toxins include indoxyl sulphate, *p*-cresyl sulphate, indole-3 acetic acid, TMAO, and phenylacetylglutamine; these are found to associate with cardiovascular diseases, mortality in CKD, and other end-organ toxicity.

Indoxyl sulphate and indole-3 acetic acid are produced by dietary tryptophan metabolism [32, 33]. Tryptophan is metabolized into indole by tryptophanase of intestinal bacteria such as *Escherichia coli*; after intestinal absorption, indole is sulphated to indoxyl sulphate in the liver. Indoxyl sulphate is normally excreted in urine; it cannot be efficiently cleaned by conventional hemodialysis because of its high binding affinity for albumin [34].

*p*-Cresol/*p*-cresyl sulphate is produced from phenylalanine and tyrosine catabolism by anaerobic gut bacteria. *p*-Cresol is conjugated by intestinal microbes to *p*-cresyl sulphate and *p*-cresyl glucuronide. *p*-Cresyl sulphate is a toxin due to its high circulated concentration and biochemical impact in the body [35]. *p*-Cresol is conjugated also in the liver as well as it can compete with xenobiotics that have either similar structure or moiety in their skeletal structure, which in turn can affect their corresponding pharmacokinetic/pharmacodynamic profiles (including toxicity/adverse effects) [25].

TMAO is a gut-derived toxic metabolite from bacterial metabolism of quaternary amines that include betaine, l-carnitine or phosphatidylcholine that release trimethylamine [36]. Trimethylamine is absorbed and converted to TMAO by flavin monooxygenase enzymes in the liver. Unlike the protein-bound toxic metabolites such as indoxyl sulphate and *p*-cresyl sulphate, TMAO can be efficiently removed by dialysis.

Phenylacetylglutamine is another colonic microbial product, produced from phenylalanine fermentation. Microbes metabolize phenylalanine to phenylacetic acid, which undergoes glutamine conjugation to form phenylacetylglutamine. Like TMAO, it is dialyzable. The uremic state has been demonstrated to induce changes in gut microbiota. Despite no significant differences in total amount of microorganisms, an erosion of the aerobic bacteria by the anaerobic bacteria (especially *Lactobacillus* and *Bifidobacterium*) has been described [37, 38]. The increase in anaerobic bacteria promoted the degradation of nitrogen compounds in deteriorative uremic state [39].

### Dysbiosis of gut microbiota and the dysfunction of gut-epithelial barrier

The intestinal epithelium is a single layer of columnar epithelial cells that separates intestinal lumen from the underlying lamina propria [40]. It plays an important role in nutrient absorption, and is a natural barrier that prevents or inhibits systemic translocation of pathogens and antigens [40]. These cells are bound together by tight junctions, forming a multifunctional complex as a seal between adjacent epithelial cells [40]. Probiotic bacteria improve intestinal epithelial barrier function in both animals and human [41]. Treating human epithelial cell monolayers with metabolites from *Bifidobacterium* infantis resulted in increase of tight junction proteins ZO-1 and occludin yet decrease of claudin-2, henceforth the selectivity of tight junction was indicated [42]. Moreover, commensal bacteria help maintain the intestinal epithelial barrier by suppressing intestinal inflammation [43].

First, urea is hydrolysed by urease to yield ammonia and carbamate that decomposes spontaneously to yield a second molecule of ammonia and bicarbonate. Ammonia then undergoes an acid–base reaction with water to yield ammonium hydroxide. Blood urea diffuses into the gut lumen and was metabolized by bacteria-derived urease, producing NH_3_ that is hydrolyzed into NH_4_OH, which erodes the epithelial barrier [38, 44]. This further stimulated influx of leukocytes, which evoked the second mechanism whereby local inflammation and cytokine production induced retraction and endocytosis of the transcellular tight junction proteins (claudins and occludin) [45]. As mentioned above, SCFA from gut bacteria was an important nutrient source for enterocytes, and theoretically a shift in the bacterial population jeopardized the health of the epithelial barrier.

## Gut microbiome in patients with kidney diseases

**Fig. 3:**
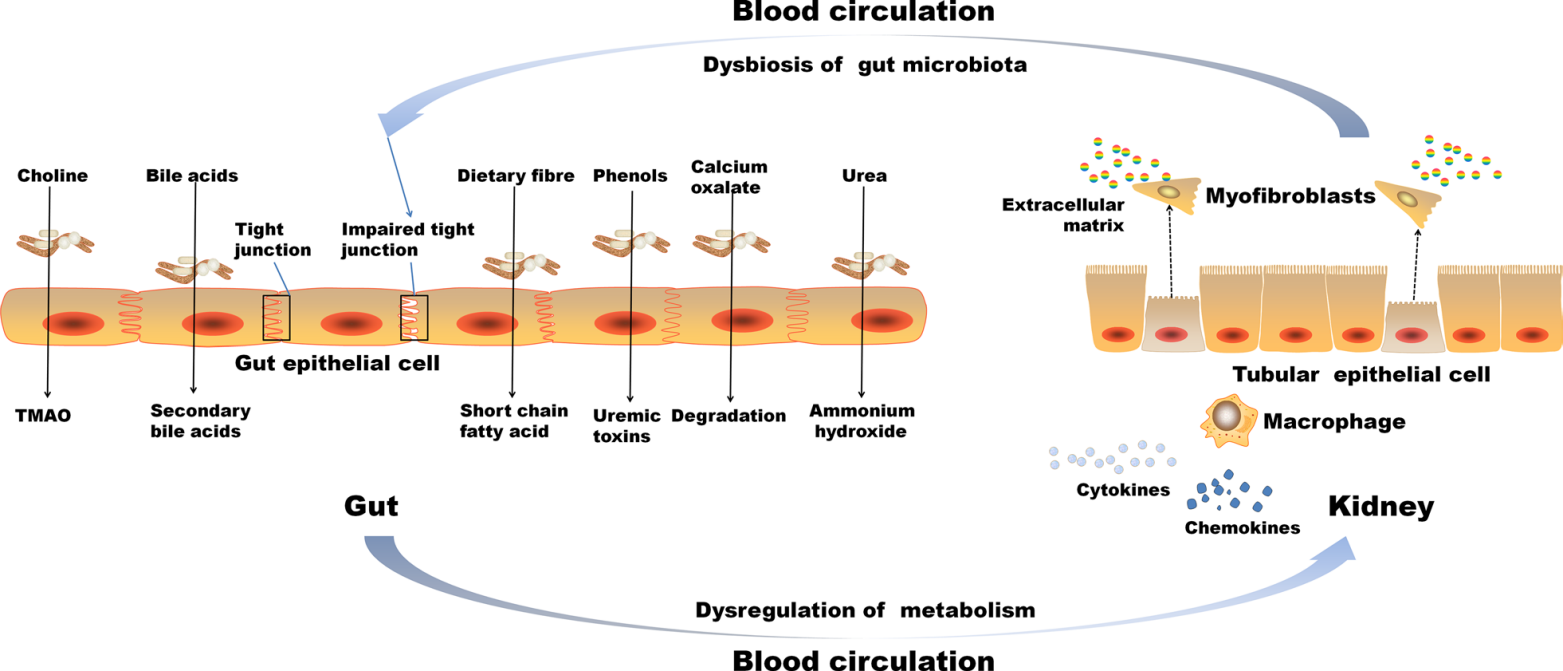
Gut–kidney axis contributes on renal injury through the dysbiosis of gut microbiota and dysregulation of endogenous metabolites. Schematic diagram presented several major metabolites involving in host-gut microbiota communication, originating from synthesis from microbial nutrient conversion, and the subsequent transport and interaction with kidney

Kidney diseases were associated with intestinal wall congestion, intestinal wall edema, slow colonic transit, metabolic acidosis, frequent use of antibiotics, decreased consumption of dietary fibers, and oral intake of iron, which impact intestinal tight junctions, lead to increased intestinal permeability, and render translocation of bacterial metabolic products across the intestinal barrier [46–49]. As a consequence, an immune response is evoked [46]. The immune response explains the systemic inflammation that contributes to deteriorating kidney disease [3, 50]. Moreover, the increased gastrointestinal urea secretion resulted in the dysbiosis of gut microbiota and increased toxic ammonia formation. Additionally, urea supplementation in drinking water contributed to alteration in bacterial gut microbiota [51]. Figure 3 presented the contribution of gut–kidney axis on renal fibrosis through the dysbiosis of gut microbiota and dysregulation of endogenous metabolites.

### Gut microbiota in CKD

Increasing evidence suggests that the gut microbiome was altered in patients with CKD. Approximately, 190 microbial operational taxonomic units (OTU) were significantly different in abundance when the gut microbiome of patients with end-stage renal disease (ESRD) was compared with healthy controls [52]. The lower numbers of *Lactobacillaceae* and *Prevotellaceae* families (both are considered normal colonic microbiota) and 100 times higher *Enterobacteria* and *Enterococci* species (which are normally present in lower proportion) were determined in CKD patients [52]. The quantity of aerobic bacteria, including the *Enterococci* and *Enterobacteria* species, was higher in patients with ESRD than in healthy controls [53]. Dysbiosis of gut microbiota in patients with CKD contributed to elevated uremic toxin concentration which in turn promoted CKD progression [54, 55]. Gut microbiota imbalance in CKD occurred both quantitatively and qualitatively, is frequently accompanied with increase in *Lachnospiraceae*, *Enterobacteriaceae* and certain *Ruminococcaceae*, and decrease in some *Prevotellaceae*, *Bacteroidaceae* and particular *Lactobacillus* and *Bifidobacterium* species [56]. The absolute quantity of total bacteria was significantly reduced in ESRD patients. *Prevotella* was prevalent in healthy controls whereas *Bacteroides* was enriched in ESRD patients. The butyrate producing bacteria, including *Roseburia*, *Faecalibacterium*, *Clostridium*, *Coprococcus*, and *Prevotella*, were reduced in ESRD patients [57].

Our studies further indicated that the dysregulations of oxidative stress and inflammation were associated with the perturbations of serum amino acid, lipid, purine and lipid metabolisms in CKD [58, 59], which are associated with the metabolism of gut microbiota. Additionally, recent clinical studies have shown that blood triglycerides and HDL-cholesterol level and predict metabolic response to diet and drug were associated with gut microbiota composition [60]. Impaired renal function and dysbiosis of gut microbiota contributed to increased TMAO in CKD patients [61]. Faecal samples from CKD patients and healthy controls were administrated to antibiotic-treated C57BL/6 mice, and the mice that received gut microbiota from CKD patients had significantly higher plasma TMAO and different gut microbiota composition than the comparative mice [61]. Besides, ammonia was metabolized from urea by microbial urease. Ammonia could cause a massive disruption of the intestinal epithelial barrier structure and function, leading to the translocation of gut-derived uremic toxins, antigens, endotoxin, and intestinal microbial organisms/products into circulation [44, 62, 63]. Indoxyl sulphate and *p*-cresyl sulphate were associated with increased inflammatory biomarkers in stage 3–4 CKD patients, such as glutathione peroxidase and interleukin-6 [64]. Another study revealed that 19 microbial families that were dominant in ESRD patients, 12 possessed urease (*Alteromonadaceae*, *Clostridiaceae*, *Cellulomonadaceae*, *Dermabacteraceae*, *Halomonadaceae*, *Enterobacteriaceae*, *Methylococcaceae*, *Moraxellaceae*, *Micrococcaceae*, *Polyangiaceae*, *Xanthomonadaceae*, and *Pseudomonadaceae*), 5 possessed uricase (*Cellulomonadaceae*, *Micrococcaceae*, *Dermabacteraceaea*, *Xanthomonadaceae* and *Polyangiaceae* families), and 3 possessed indole and *p*-cresyl-forming enzymes (i.e. tryptophanase possessing families: *Clostridiaceae*, *Verrucomicrobiaceae*, and *Enterobacteriaceae*) [65]. *Prevotellaceae* and *Lactobacillaceae*, the two families that possess SCFA (butyrate) forming enzymes, were amongst the four microbial families that were depleted in ESRD patients [65].

Based on metabolomics, our previous studies demonstrated that the perturbations of amino acid, lipid, purine metabolisms in serum [66–70] as well as bile acid and phospholipid metabolisms in faeces are related to CKD rats [71, 72]. The disruption of the intestinal barrier in CKD led to translocation of bacteria-derived uremic toxins into the systemic circulation, thus inducing inflammation and leukocyte stimulation. Using metabolomics methods, our previous studies demonstrated that the dysregulations of oxidative stress and inflammation were associated with the perturbations of serum amino acid, methylamine, purine and lipid metabolisms in patients with CKD [31, 73–75].

### Gut microbiota in patients on hemodialysis and peritoneal dialysis

By replacing kidney excretory function, dialysis is intended to eliminate the symptom complex known as the uremic syndrome. Hemodialysis has made survival possible for more than a million people throughout the world who have ESRD with limited or no kidney function [76, 77]. Through metabolomics methods, our previous studies indicated that the uremic toxins and waste products in hemodialysis removed a large number of identified and as-yet unidentified metabolites [78]. Phylogenetic microarrays analysis demonstrated that the gut microbiome of ESRD patients with hemodialysis and compared them with healthy individuals, showing an increase in *Proteobacteria* (primarily *Gammaproteobacteria*), *Actinobacteria* and *Firmicutes* (especially subphylum *Clostridia*) [52]. However, hemodialysis patients showed higher inflammatory biomarkers and uremic toxins than non-dialysis patients [79]. Interleukin-6 and MCP-1, two inflammatory biomarkers, were positively correlated with indoxyl sulphate and *p*-cresyl sulphate [79]. The reduced levels of uremic toxins resulted in the decreased expression of inflammatory biomarkers [80]. The gut microbiome in pediatric patients undergoing hemodialysis was compared against those of healthy individuals [81]. *Bacteroidetes* was significantly increased while *Proteobacteria* was significantly decreased in hemodialysis patients compared with healthy individuals [81]. Additionally, fecal analysis demonstrated that dialysis patients showed decreased number of bacteria that were able to produce the SCFA butyrate [65].

One study described a decrease in gut *Firmicutes* and *Actinobacteria*, especially *Bifidobacterium catenulatum*, *Bifidobacterium bifidum*, *Bifidobacterium longum*, *Lactobacillus plantarum* and *Lactobacillus paracasei* in peritoneal dialysis patients [82]. In general, patients with CKD exhibited lower intestinal colonization of *Bifidobacterium* and *Lactobacillus* species [56]. Therefore, reduced populations and diversity of *Lactobacillus* and *Bifidobacterium* in peritoneal dialysis patients were associated with several adverse effects. Pediatric peritoneal dialysis patients showed a relative lower abundance of gut bacteria within the *Firmicutes* and *Actinobacteria*, whereas the *Proteobacteria* were significantly increased [81]. The increased *Proteobacteria* (iron oxidizing bacteria) was associated with the oral iron supplementation in peritoneal dialysis patients. Additionally, peritoneal dialysis patients enhanced intestinal absorption of glucose from the peritoneal dialysis dialysate that promoted glucose fermentable bacteria *Enterobacteriaceae* [81]. Considering the translocation of gut microbiota to the peritoneal cavity, it was presumed that the increase of *Enterobacteriaceae* was responsible for peritonitis development in peritoneal dialysis patients, since *Enterobacteriaceae* family accounted for up to 12% of all peritonitis episodes in these patients [83].

### Gut microbiota in IgAN

Since immunoglobulin A (IgA) is widely found in gut mucosal immune system, dysbiosis of gut microbiota plays a role in the pathogenesis of IgAN [55]. Chronic bacterial infections and dysbiosis of gut microbiota enhanced epithelial cells to secrete B cell activating factor and proliferation-inducing ligand that speeded up overproduction of IgA. Additionally, dysbiosis of gut microbiota were found in IgAN [55]. Exclusive differences in gut microbiota and metabolome composition were investigated in patients with IgAN and healthy controls [84, 85], and the gut microbiota and urinary metabolites (including free amino acids and organic volatile metabolites) were significantly altered between patients with progressor and non-progressor IgAN [86]. It was speculated that the elevated serum free amino acids contributed to IgAN pathology where possibly associated the lowered absorption of gastrointestinal proteins, which presumably enhanced microbial proteolysis, changed microbiota, and contributed to elevated fecal *p*-cresol level. The potential link between bacterial lipopolysaccharides and hypogalactosylation of IgA existed. Bacterial lipopolysaccharide could stimulate a systemic inflammatory response and lipopolysaccharides was involved in the hyperproduction and hypogalactosylation of IgA1, the important pathogenesis involved in IgAN [87].

### Gut microbiota in nephrolithiasis

Nephrolithiasis is a complex disease that could be caused by genetic and different environmental factors. Kidney stones are small deposits that build up in the kidneys, made of calcium, phosphate and other components of foods. Hyperoxaluria is an important risk factor for the appearance of nephrolithiasis, since 75% of kidney stones contain calcium oxalate [88]. Since human body relies mainly on gut microbiota for oxalate homeostasis, *Oxalobacter formigenes* has attracted attention in medicine [89]. The *Oxalobacter formigenes*, as an oxalate degrader bacterium in the intestinal tract, showed health benefits through the homeostasis of oxalic acid [90]. An inverse relationship was demonstrated between recurrent renal stones and intestinal colonization with *Oxalobacter formigenes*, which reduced the oxalate concentration that was available for absorption at constant rates in the intestine. *Oxalobacter formigenes* could lower oxalate excretion in urine and protect against formation of calcium oxalate kidney stones [91, 92]. Besides, gut microbiome participated in the pathophysiology of kidney stone formation [92]. Patients with nephrolithiasis possessed a unique gut microbiota compared with healthy controls [93]. *Bacteroides* spp. was more abundant in kidney stone formers where *Prevotella* spp. was more abundant in the healthy controls [93].

In addition, cyanuric acid was produced from melamine in gut by microbial transformation and it served as an integral component of the kidney stones responsible for melamine-induced renal toxicity in rats [94]. *Klebsiella* was subsequently identified in faeces and could convert melamine to cyanuric acid directly. Rats colonized by *Klebsiella* terrigena displayed exacerbated melamine-induced nephrotoxicity [94]. Currently available data supported that manipulation of gut bacteria may provide a novel therapy in patients with kidney stone in the future.

### Gut microbiome in hypertension

Patients with elevated systolic blood pressure and CKD revealed altered bacterial composition and decreased bacterial richness [95]. The abundance of the gut microbes, *Firmicutes* and *Bacteroidetes*, is associated with increased blood pressure in several models of hypertension [96]. It has been reported that major component of the olfactory pathway in kidneys, Olfr78, was an olfactory receptor expressed in the renal juxtaglomerular apparatus, where it mediated renin secretion in response to SCFAs. SCFAs were fermentation end-products by the gut microbiota and were absorbed into the circulation [97]. Another possible link between the gut microbiota and hypertension was the gut microbiota metabolism of choline and phosphatidylcholine, which metabolized trimethylamine to TMAO. Trimethylamine is abundant in red meat and can be metabolized by intestinal microbiota of dietary l-carnitine, and further can be metabolized into TMAO and expedited atherosclerosis in mice [98].

### Gut microbiome in acute kidney injury

Recently, several studies indicated that intestinal microbiota can regulate AKI. One possible mechanism was the renoprotective action of SCFAs against ischaemia–reperfusion injury in models. SCFAs with anti-inflammatory properties were produced by gut microbiota [99]. Treatment with three main SCFAs (acetate, propionate, and butyrate) improved renal dysfunction and reduced inflammation. Furthermore, the gut microbiota showed a wider influence and role in autoimmune kidney diseases via its immunomodulatory effects, known by its effect on polarization of T-cell subsets and natural killer cells [32].

## Probiotic, prebiotic and synbiotic interventions to attenuate gut microbiome disturbances in kidney diseases

The use of probiotics and prebiotics are common therapeutics. Probiotics are living organisms ingested through food or supplements that could promote the health of the host. Probiotics are composed of living bacteria, such as *Lactobacilli*, *Streptococci* and *Bifidobacteria* species, that could alter gut microbiota and affect the inflammatory state to produce a less pathogenic microflora and thus lowered generation of uremic toxins. A pilot multinational trial in patients with CKD stages 3 and 4 showed significantly decreased blood urea and improved life quality after treatment with the Renadyl formulation of *Lactobacillus acidophilus, Streptococcus thermophilus* and *Bifidobacterium longum* over 6 months [100]. However, the follow-up randomized controlled trial in 22 patients failed to lower plasma uremic toxins and did not improve life quality [101]. The few benefits with probiotics could be explained by persistent uremia-induced alterations in gut biochemical milieu and dietary and medicinal regimens which led to an unfavorable milieu for the symbiotic microbiota [102]. To address this deficit, one trial investigated the combination of probiotic and prebiotic therapies over a course of 6 weeks in pre-dialysis CKD patients, and showed lowered serum *p*-cresyl sulphate and gut microbiome alterations [103]. Therefore the choice of probiotic microbe is important. Inclusion of bacteria that expressed urease with the intention to metabolize gut urea caused the increased downstream products NH_3_ and NH_4_OH and promoted intestinal wall inflammation [102, 104].

Prebiotics are non-digestible carbohydrates that selectively stimulate the growth and activity of beneficial gut bacteria in colon, such as *Bifidobacteria* [105]. Prebiotics promote the growth of *Bifidobacteria* and *Lactobacilli* species at the expense of other groups of bacteria in the gut [105]. Prebiotic oligofructose-enriched p-inulin also regulated weight loss, inhibited inflammation, and improved metabolic function [105]. Serum *p*-cresol and indoxyl sulphate are lowered by the oral intake of p-inulin in hemodialysis patients [106]. However, feeding uremic rats treated with amylose maize-resistant starch could improve creatinine clearance and lower inflammation and renal fibrosis [107]. The semipurified low-fiber diet or a high-fiber diet significantly improved metabolomes in serum, urine and intestinal fluid accompanied by lowering dysbiosis of gut microbiota [108]. Resistant starches transited to the colon undigested and were metabolized by bacteria to SCFA which were important nutrients to enterocytes. The supplementation of oligofructose-inulin or resistant starch significantly lowered circulating indoxyl sulphate and *p*-cresyl sulphate in hemodialysis patients [106, 109].

Synbiotics is the combination of prebiotic and probiotic treatments. Treatment with Probinul neutro, synbiotic treatment, showed decreased total plasma *p*-cresol without improvement of gastrointestinal symptoms in 30 stage 3–4 CKD patients for 4 weeks [110]. The SINERGY trial showed a decrease in serum *p*-cresyl sulphate but not in indoxyl sulphate and a favorable change in stool microbiome in 37 stage 4–5 CKD patients [103]. Treatment with the combination of *Lactobacillus casei* strain Shirota and *Bifidobacterium breve* strain Yakult plus galacto-oligosaccharides showed a significant decrease of serum *p*-cresol and improvement of stools quantity and quality in nine hemodialysis patients for 2 weeks [39]. More recently, a multicenter study in 42 hemodialysis patients showed an improvement of gastrointestinal symptoms and decreased C-reactive protein after 2 months’ treatment [111].

## Concluding remarks

Increasing evidence has demonstrated that a bidirectional relationship existed between host and gut microbiome in patients with various kidney diseases. There is an urgent need for more studies to further characterize the gut microbiome in kidney diseases and explore the relationship between different kidney diseases and the gut microbiome. Intestinal inflammation and epithelial barrier breakdown accelerate systemic translocation of the bacterial-derived uremic toxins including indoxyl sulphate, *p*-cresyl sulphate, and TMAO, and cause oxidative stress injury to the kidney, cardiovascular and endocrine systems. Recently, the study of the gut–kidney axis has opened up novel therapeutic avenues for the management of inflammation, kidney injury and uremia to prevent adverse outcomes in CKD patients. Multiple promising interventions were exerted to reverse gut microbiota imbalance and slow the progression of kidney diseases. The probiotics or their byproducts have been employed to develop innovative signaling-targeted interventions which outperform traditional drugs with obvious side effects. Selecting specific probiotic species with well-known metabolic functions could alleviate various disease states. For example, *Streptococcus thermophiles* can be used to reduce urea from uremia. Future attention and examination of these interventions are required to bring the knowledge of the microbiota into practical benefits of CKD patients. However, interventions need to be further examined in large trials before they can become a primary therapy for patients with kidney diseases.

The metagenomics and metabolomics have been used to investigate the function of key low-molecular-weight endogenous metabolites derived from the gut microbiome in kidney diseases. Understanding the metabolic capabilities of gut microbiota is very important in elucidating their functions on health and disease. Although 16S rRNA sequencing analysis was employed to conveniently survey the composition and structure of gut microbiome, the information on their metabolite effects were limited by the incomplete knowledge in bacterial genomic databases. Metagenomic sequencing mines more knowledge of the existent genes, but the functions of most of these genes remain unknown. KEGG and MetaCyc are the most comprehensive databases for linking orthologous gene groups to reactions and metabolites. To achieve more effective combination of microbiome and metabolome for understanding gut microbial metabolisms in the kidney disease context, advanced multi-omic integration methods need to be developed. To further our understanding of the functional potential of host-associated gut microbiota, we can fill the gaps of the aforementioned databases through genome sequencing, untargeted biochemistry, and functional studies. Thus, even with these enormous challenges, increasing studies have found key microbes and their enzymes/metabolites as potential targets of medical interventions in the context of kidney diseases. With improved understanding of the metabolic interplay between the microbiome and the host, novel prebiotics and probiotics can be explored, and personalized treatment of CKD that utilize knowledge of gut microbiome and their interactions with the host will become feasible.

## Abbreviations

AKI: acute kidney injury
CKD: chronic kidney disease
ESRD: end-stage renal disease
IgA: immunoglobulin A
IgAN: immunoglobulin A nephropathy
OTU: operational taxonomic units
rRNA: ribosomal RNA
SCFA: short-chain fatty acids
TMAO: trimethylamine N-oxide
